# A Quantitative Approach to Determine Hydrophobe Content of Associating Polyacrylamide Using a Fluorescent Probe

**DOI:** 10.3390/molecules28104152

**Published:** 2023-05-17

**Authors:** Ziyang Su, Yu Zhang, Weidong Liu, Ruijing Han, Xuezhi Zhao, Xiaohuo Shi, Xingyu Lu, Yan Zhang, Yujun Feng

**Affiliations:** 1 Polymer Research Institute, State Key Laboratory of Polymer Materials Engineering, Sichuan University, Chengdu 610065, China; szy@stu.scu.edu.cn (Z.S.); zhaoxz@scu.edu.cn (X.Z.); 2 Research Institute of Petroleum Exploration & Development, PetroChina Company Limited, Beijing 100083, China; zhangyu01@petrochina.com.cn (Y.Z.); lwd69@petrochina.com.cn (W.L.); hanrj_18@petrochina.com.cn (R.H.); 3 Instrumentation and Service Center for Molecular Sciences, Westlake University, Hangzhou 310024, China; shixiaohuo@westlake.edu.cn (X.S.); luxingyu@westlake.edu.cn (X.L.)

**Keywords:** hydrophobically associating polymer, hydrophobe content, fluorescence spectra, scaling relationship

## Abstract

Hydrophobically associating polymers have found widespread applications in many domains due to their unique rheological behavior, which is primarily dictated by the hydrophobe content. However, the low fraction of hydrophobic monomers in polymers makes this parameter’s precise and straightforward measurement difficult. Herein, a variety of hydrophobically associating polyacrylamides (HAPAM) with different alkyl chain lengths (*L*) and hydrophobic contents ([*H*]) were prepared by post-modification and accurately characterized by ^1^H NMR spectroscopy. The maximal fluorescence emission intensity (*I*) of 8-anilino-1-naphthalenesulfonic acid, which is sensitive to hydrophobic environments, was then detected in those polymer solutions and shown as a ratio to that in the polymer-free solution (*I*_0_). It was found that *I*/*I*_0_ for 0.5 wt% HAPAM can be scaled versus *C*_H_, which is a variate related to both *L* and [*H*], as *I*/*I*_0_ = 1.15 + 1.09 × 10^8^*C*_H_^3.42^, which was also verified to be applicable for hydrophobic associating hydrolyzed polyacrylamide (HHAPAM). This relationship provides a handy method for determining the hydrophobic content of hydrophobically associating polymers, particularly for field applications.

## 1. Introduction

Hydrophobically associating polymers refer to a class of polymers that carry a small proportion of hydrophobic groups in a hydrophilic backbone. Above a critical concentration, the hydrophobic moieties from different main chains bunch together and thus form a transitory three-dimensional network [1], causing a significant rise in solution viscosity. This distinct rheological behavior enables hydrophobically associating polymers to have a wide range of applications in petroleum [2], coatings [3], sewage treatment [4], biomedicine [5], and other domains. Nevertheless, because of the low number of hydrophobic lateral groups, characterizing these polymers’ structure and hydrophobic content ([*H*]) remains a considerable challenge.

In general, the amount of hydrophobic segments required to balance the water solubility of polymers is less than 2 mol% [6], and this value even lowers to 1 mol% as the alkyl chain length (*L*) of the hydrophobic monomers grows [7,8]. Most instruments are incapable of analyzing such low contents [9]. Early studies [10,11] assumed that the [*H*] of the final polymer was roughly equal to the initial feeding ratio of hydrophobic monomers. This claim was soon disproved because the micelle polymerization strategy commonly used for hydrophobic associating polymers readily leads to drift and inhomogeneity in the final polymer composition [12]. Another approach to calculating [*H*] is to measure the UV absorption spectrum of the polymer solution in the presence of a chromophore on the hydrophobic lateral chain [9]. However, many hydrophobic monomers do not have a chromogenic group in their molecular structure, which limits the extension of the above method.

Furthermore, attempts have been made worldwide to directly detect the [*H*] of hydrophobic associating polymers using spectral techniques. For instance, Feng et al. [6] used ^1^H NMR to calculate the [*H*] of hydrophobically associating polyacrylamide (HAPAM), and the results ranged from 0.5 mol% to 1.0 mol%, which are consistent with the expected experimental values. Even though this technique can accurately examine [*H*] within a certain range, its complex sample preparation, picky testing, and high equipment requirements render it unsuitable for field use. Additionally, the fluorescent probe, e.g., pyrene, was employed to label the hydrophobic microdomain in the polymer solution, allowing the hydrophobicity of the polymer to be reflected in the fluorescence emission spectrum of the solution [13,14,15]. This method only provides some qualitative information on hydrophobic incorporation. Hence, a simple and quantitative approach to determining [*H*] is urgently required to be developed, particularly for industrial polymer products in field applications.

In this work, as model polymers, a series of hydrophobically associating polyacrylamides (HAPAMs) with various *L* or different [*H*] was synthesized by post-modification and accurately characterized via ^1^H NMR. These polymer solutions were then blended with 8-aniline-1-naphthalene sulfonic acid (ANS), whose fluorescence emission intensity represents the hydrophobicity of polymers. In this way, the relationship between fluorescence intensity and [*H*] could be investigated based on the ratio (*I*/*I*_0_) of all model polymers’ maximum fluorescence emission intensity to pure ANS solution. Eventually, the correlation equations were validated using several hydrophobically associating hydrolyzed polyacrylamide (HHAPAM) products from different manufacturers.

## 2. Results and Discussion

### 2.1. Synthesis and Structural Characterization of HAPAM

The preparation of HAPAM by post-modification was first reported by Deguchi and Lindman [16] and later further improved by Feng [6]. Differing from micelle polymerization, the hydrophobic groups of HAPAM are randomly distributed, which results in a uniform composition. 

**Figure 1:**
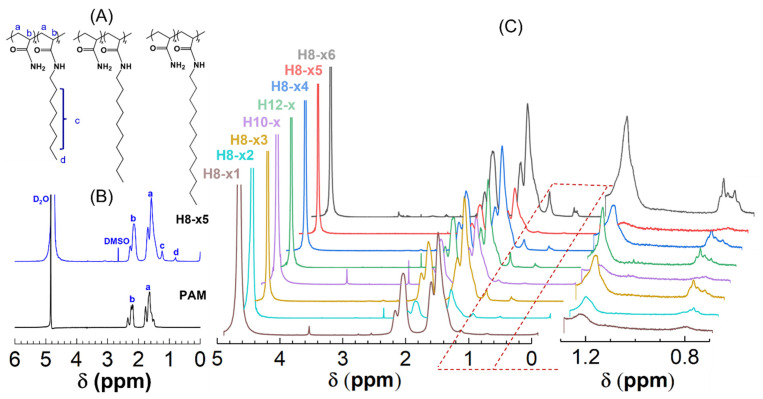
(**A**) Structural formula of H-series polymers; (**B**) ^1^H NMR spectra of H8-×5 and PAM; and (**C**) ^1^H NMR spectra of H-series hydrophobically associating polymers in D_2_O. (a, b, c and d refer to the proton peak in the corresponding groups).

Meanwhile, according to Candau et al. [10], at least six to eight carbons of an alkyl chain were requested for the hydrophobic interaction, and the longer the side chain, the less soluble the polymer is. After comprehensive consideration, we chose octyl/decyl/dodecyl chains as the target hydrophobic groups. Hence, a series of hydrophobically associating polymers with octyl/decyl/dodecyl chains was synthesized in this way, and their chemical structures are shown in Figure 1A.

As stated in the introduction, NMR is a commonly used technique to characterize copolymer composition. Even though only a few alkyl bromide groups were added to the feed in our work, it is feasible to quantify the hydrophobic content of H-series polymers because their hydrophobic lateral chain contains characteristic protons in the terminal methyl and methylene groups.

Taking one of H8-x as an example, Figure 1B compares the ^1^H NMR spectra of H8-x and its unmodified counterpart, PAM. One can find that the protons of the methylene (−CH_2_−) and methenyl (−CH−) groups in the backbones of both polymers are evident in ranges of 1.3~1.7 ppm and 1.9~2.2 ppm, respectively. As expected, the proton peaks at 1.2 and 0.8 ppm are only observed for HAPAM, which are attributed to the methylene of octyl groups except for the α-position methylene next to the amide group [−(CH_2_)_n_−] and terminal methyl group (−CH_3_) of the hydrophobe. Similar observations are also obtained for other H-series polymers (Figure 1C).

Hence, the ^1^H NMR spectra confirm that the alkyl chain was successfully grafted into PAM.

Moreover, the peaks at 0.8 can be used to calculate the hydrophobic content from their integral areas based on the following equation [17]:

(1) $$\frac{S_{d}}{S_{a}{+S}_{b}{+S}_{c}}=\frac{3y}{3x+15y}$$

(2) x+y=100

where *S*_i_ (i = a, b, c, d) stands for the integrated areas of the proton peak in the corresponding groups, and *x* and *y* refer to the molar contents of the AM and hydrophobe units, respectively. In this way, the hydrophobic content of all H-series polymers is acquired as listed in Table 1.

### 2.2. Determination of Hydrophobic Content of HAPAM with Fluorescence Spectra

To quantitatively scale the relationship of hydrophobic content, a fluorescence probe method was employed to analyze the fluorescence intensity of polymers of known structure. It is well-recognized that ANS is a water-soluble (0.1 g/5 mL) fluorescent dye, yet its aqueous solution only emits slight fluorescence; as the polarity of the microenvironment of the ANS molecules decreases, a much stronger fluorescence intensity is achieved, as well as an obvious blue shift of the emission wavelength at maximum fluorescence intensity. The prevailing explanation for this phenomenon [18] suggests that it is related to solvent relaxation and the dipole moments of the different states of the ANS molecule. Therefore, ANS is harnessed here to label the hydrophobic microenvironment of HAPAM aqueous solutions at a fixed concentration of 0.5 wt%.

#### 2.2.1. Fluorescence Spectra Results

**Figure 2:**
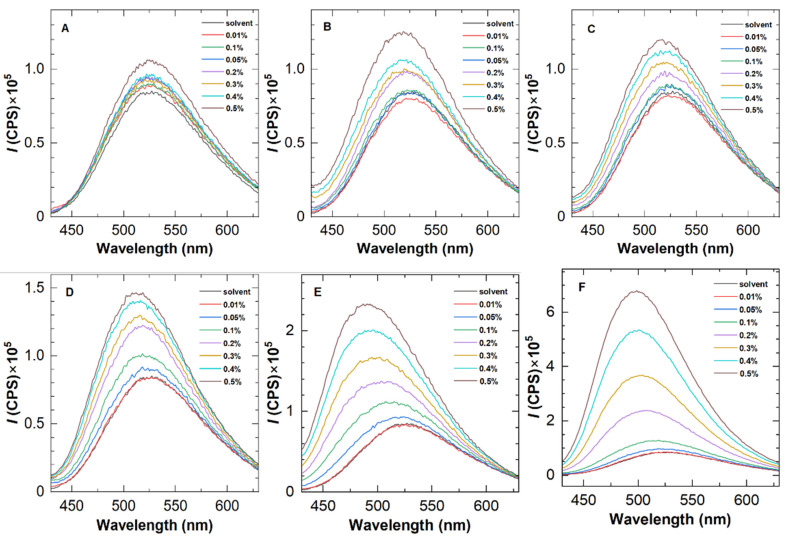
Fluorescence emission spectra of five representative HAPAM polymers at 25 °C with an excitation wavelength of 415 nm. The concentration of ANS was 0.05 wt%. (**A**–**F**) refer to PAM, H8-0.5, H10-0.5, H12-0.5, H8-1.0, and H8-1.5 in sequence.

Firstly, the availability of ANS was evaluated by fluorescence spectra using five representative polymers, i.e., H8-0.5, H8-1.0, H8-1.5, H10-0.5, and H12-0.5. Figure 2 depicts their fluorescence emission spectra at different polymer concentrations, with an excitation wavelength of 415 nm, which reveals two distinct features. On the one hand, as the polymer concentration improves to a certain value, the fluorescence intensity increases clearly, confirming the existence of a hydrophobic environment in the solution and indicating the occurrence of hydrophobic association. At the same polymer concentration, H8-1.0 and H8-1.5 show the greatest fluorescence enhancement among the five polymers, demonstrating a positive correlation between the fluorescence intensity and hydrophobic content.

**Figure 3:**
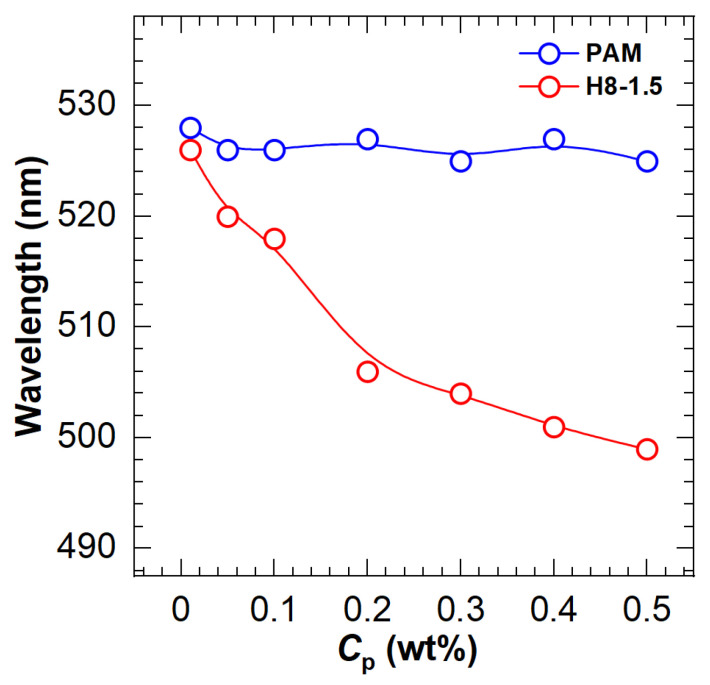
Variation of maximum emission wavelength with polymer concentration (*C*_p_) for PAM and H8-1.5.

On the other hand, blue shifts were found in the fluorescence spectra of HAPAM. According to Siano et al. [19], the blue shift of dye in polymer solutions explains fluorescent probe solubilization in hydrophobic microdomains. For the same polymer, the blue shift with increasing concentration demonstrates the enhancement of the hydrophobic microregion. To better observe the blue shift, Figure 3 plots the wavelength at the maximum fluorescence emission peak as a function of polymer concentration for an example HAPAM (H8-1.5) and PAM. In the case of PAM, the wavelengths are almost 526 nm over the entire range of polymer concentration. For H8-1.5, however, the maximum emission wavelength decreases step by step as the polymer concentration (*C*_p_) increases and is 499 nm at a *C*_p_ of 0.5%. These findings demonstrate that ANS can be used to characterize the hydrophobic microregions of HAPAM.

**Figure 4:**
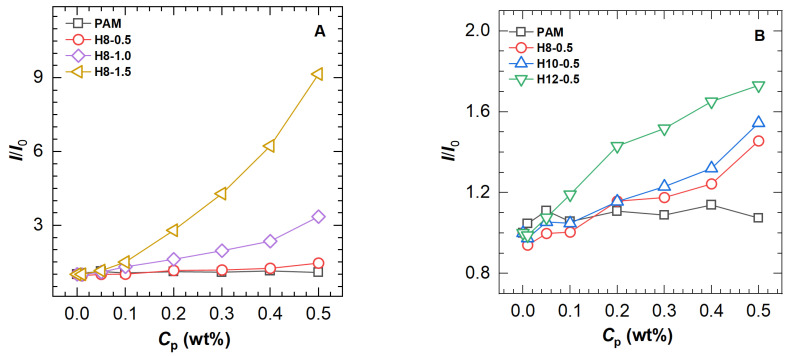
The ratio (*I*/*I*_0_) of maximum fluorescence intensity of polymers to that of solvent as a function of polymer concentration (*C*_p_) for PAM and HAPAM (**A**) with different hydrophobe content and (**B**) with different hydrophobic chain length.

To further analyze the differences among the polymers, the maximum fluorescence intensity of the polymer solutions was compared to that of a pure ANS solution to obtain a ratio *I*/*I*_0_, which is plotted as a function of polymer concentration in Figure 4. The *I*/*I*_0_ value of the unmodified PAM sample has no response to the polymer concentration increase, which is in agreement with the change in maximum emission wavelength and can be interpreted as the immunity of ANS to amide functions in aqueous solution. Nevertheless, a similar profile is only observed at low concentrations for all HAPAM polymers. When the polymer concentration is above 0.1 wt%, the *I*/*I*_0_ value increases drastically, indicating a gradual growth of hydrophobic microdomains.

Comparing polymers (H8-0.5, H8-1.0, and H8-1.5, Figure 4A with different [*H*] values, the amplitude of *I*/*I*_0_ enhancement increases with increasing [*H*]. A similar trend is found for three polymers (H8-0.5, H10-0.5, and H12-0.5, Figure 4B with different *L* values, i.e., the higher the *L* value, the stronger the *I*/*I*_0_ enhancing the ability of the polymer. For the same type of polymer, long-chain hydrophobic groups tend to be more hydrophobic than short-chain ones and thus more likely to form hydrophobic microdomains. These findings suggest that both [*H*] and *L* values would affect the fluorescence intensity of ANS in the HAPAM polymer solution.

#### 2.2.2. Scaling Relationship

From Section 3.2, we conclude that the ANS-based fluorescence probe method is indeed useful for the hydrophobicity detection of HAPAM, and the higher the hydrophobic content, the stronger the hydrophobicity-induced fluorescence intensity enhancement of the polymer/ANS solution. In this section, we further quantify the relationship between fluorescence intensity and [*H*] using polymers H8-x at a fixed polymer concentration. Given that increasing the polymer concentration can magnify the divergence between PAM and HAPAM, a relatively high polymer concentration, i.e., 0.5 wt%, was selected.

**Figure 5:**
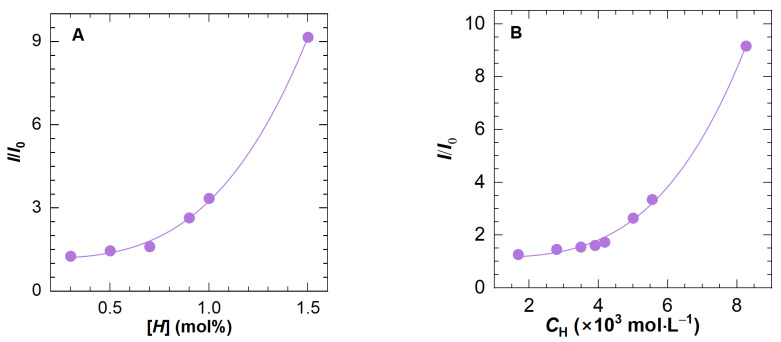
The ratio of maximum fluorescence intensity of polymer solution to that of solvent (*I*/*I*_0_) as a function of (**A**) the hydrophobic content ([*H*]) for H8-x and (**B**) the total molar concentration of −CH_2_− and −CH_3_ in the hydrophobic side chain for all HAPAM polymers. The concentration of ANS and polymer are fixed at 0.05 wt% and 0.5 wt%, respectively.

As the concentration of 0.5 wt% is obviously beyond the dilute solution region, polymer molecular chains entangle with each other, which promotes the formation of intermolecular association; in other words, the contribution of intramolecular association to the fluorescence intensity can be disregarded. Presented in Figure 5A is the *I*/*I*_0_ change of HAPAM as a function of [*H*], the hydrophobic content of polymers H8-x. An exponential curve can fit the data well. To be specific, at low [*H*] values, the change in the maximum relative fluorescence intensity is invisible, indicating that hydrophobic microdomains have not formed in large numbers. Despite the absence of microdomains, McCormick et al. [20] found that the probe still interacts with the hydrophobic segments of the expanded polymer coil. This is where the fluorescence intensity in the low [*H*] range originates from. With the gradual increase in [*H*], the hydrophobic microdomains begin to connect and form a physical network structure, thereby accelerating the dissolution of ANS and eventually leading to a sharp increase in the slope of the curve.

Based on the above-mentioned fitting curve, aquantitative equation was acquired as follows:

(3) $$I/I_{0}=1.18+2.1{\left[H\right]}^{3.3}$$

where the constant 1.18 stems from two parts: “1” represents the fluorescence intensity of the solvent, while “0.18” corresponds to the influence of environmental microviscosity on ANS fluorescence quenching. According to Edelman et al. [21], the improvement in the solution viscosity will reduce the quenching of the fluorescence probe in the solution, thereby resulting in higher fluorescence intensity. When the viscosity is increased to 10 mPa·s, however, the effect of viscosity on the fluorescence intensity of the solution will remain unchanged. In our work, the viscosities of all polymer solutions at a concentration of 0.5 wt% are much higher than 10 mPa·s, which means that the contribution of solution viscosity to fluorescence intensity can be treated as a constant. This is exactly the practical significance of “0.18” in Equation (3). With regard to the coefficient “2.1” and index “3.3”, both of them reflect the share of hydrophobic content in the fluorescence intensity of the solution and depend on the solvent environment and the type of hydrophobic group. As mentioned before, the fluorescence intensity of a polymer solution containing ANS bears on both [*H*] and *L* values. In theory, [*H*] mainly influences the amount of associative structures of HAPAM, whereas *L* affects not only the hydrophobicity of the chain itself, but also the spatial constraints on the limits of the hydrophobic microdomain of the solution. In this scenario, employing the content of alkyl groups can cover the effects of both *L* and [*H*]. Thus, to further explore a universal expression, the [*H*] of all eight HAPAMs was converted to *C*_H_ through the following equation:

(4) $$C_{H}=\frac{C_{p}/100\times 1000\times \left[H\right]\times L}{m_{A}\times \left(1-\left[H\right]\right)+m_{H}\times \left[H\right]}$$

where *C*_H_ means the total molar concentration of −CH_2_− and −CH_3_ in the hydrophobic side chain, mol/L; *C*_p_ is the polymer concentration and fixed at 0.5 wt%; [*H*] stands for the hydrophobic content, mol%; *L* is the number of carbon atoms in the alky chain of the hydrophobic segment; and *m*_A_ and *m*_H_ represent the molecular masses of acrylamide (71 g/mol) and hydrophobic units (e.g., 183 g/mol for polymers H8-x), respectively.

Figure 5B plots the correlation of *I*/*I*_0_ and *C*_H_ for eight HAPAM polymers. Likewise, the data can be well-fitted using an exponent expression as shown in Equation (5), through which a simple fluorescence spectra determination at a polymer concentration of 0.5 wt% can determine the hydrophobe content.

(5) $$I/I_{0}=1.15+1.09\times {\mathrm{10}}^{8}{C_{H}}^{3.42}$$

### 2.3. Application of Scaling Relationship to Hydrophobic Associating Polyelectrolyte

Although a quantitative relation for [*H*] measurement was obtained in Section 3.2 for nonionic HAPAM, most used hydrophobic associating polymers in industrial applications are polyelectrolytes, such as hydrophobic associating hydrolyzed polyacrylamide (HHAPAM). The presence of cationic groups in polymer chains could enhance the fluorescence intensity of ANS, as reported by Ricard et al. [14]. Thus, it is necessary to investigate whether Equation (5) and even the ANS-based fluorescence probe method are applicable to ionic hydrophobic association polymers. Four industrial HHAPAM products from various manufacturers and their reference HAPMs were used in this section. The solvent and test conditions remain the same as in Section 3.2.

According to calculations by Zhu et al. [17], an addition of inorganic salt (0.02 M) is sufficient to screen the ionic strength of a polyelectrolyte with a degree of hydrolysis (DH) of 44% at a polymer concentration of 1%. In this study, the DHs of four HHAPAM polymers were measured using the method of Chen et al. [22] (see Supplementary Materials) and are in ranges of 19 to 24%, and the salt content of the solvent was 10,000 mg/L (≈0.16 M), which can shield the ionic strength of all HHAPAM polymer solutions at *C*_p_ ≤ 0.5 wt%. That is why we choose a salt solution as a solvent for all experiments. Certainly, saline water also exists in many practical applications, especially in the petroleum industry.

**Figure 6:**
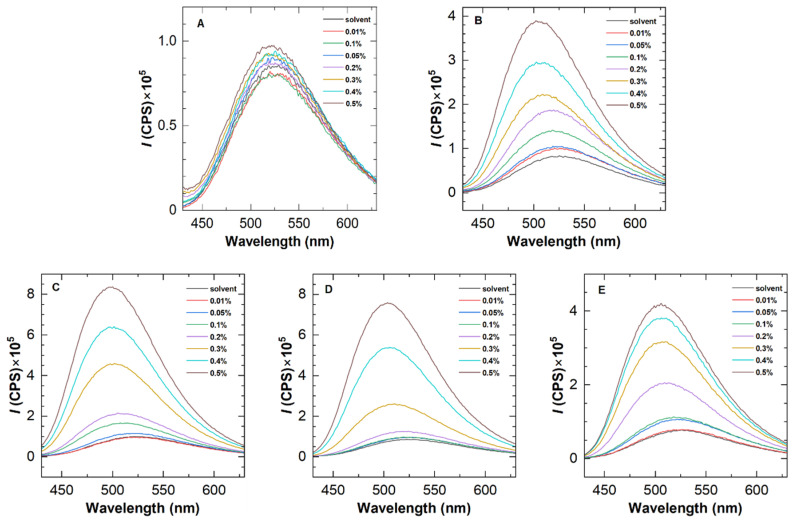
Fluorescence emission spectra of (**A**) HPAM and (**B**–**E**) four HHAPAM polymers at 25 °C with an excitation wavelength of 415 nm.

The fluorescence emission spectra of HHAPAMs and HPAM are shown in Figure 6. As with the results of HAPAM in Figure 2, a fluorescence intensity enhancement was observed for all HHAPAM polymers as the polymer concentration increased, while it was inconspicuous for the HPAM solution. Meanwhile, the maximum emission wavelength shows a blue shift of about 20 nm when the polymer concentration changes from 0.01 wt% to 0.5 wt%.

**Figure 7:**
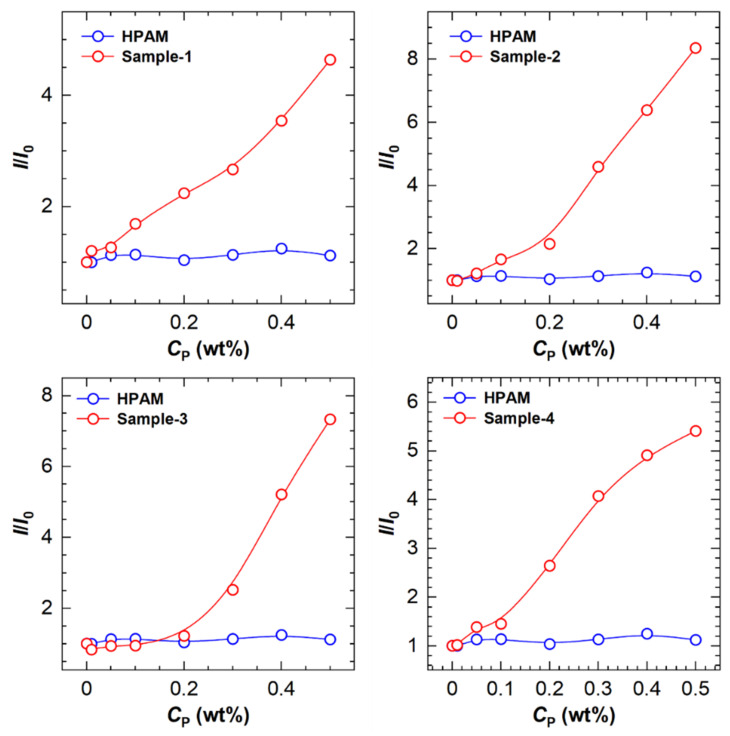
The ratio (*I*/*I*_0_) of maximum fluorescence intensity of polymers to that of solvent as a function of polymer concentration (*C*_p_) for four HHAPAM polymers.

The variation in relative fluorescence intensities (*I*/*I*_0_) with the polymer concentration is drawn in Figure 7. It was found that the critical concentrations for the transition of fluorescence intensity are in the range of 0.05 wt% to 0.2 wt%, above which the hydrophobic microregions are quickly formed.

**Figure 8:**
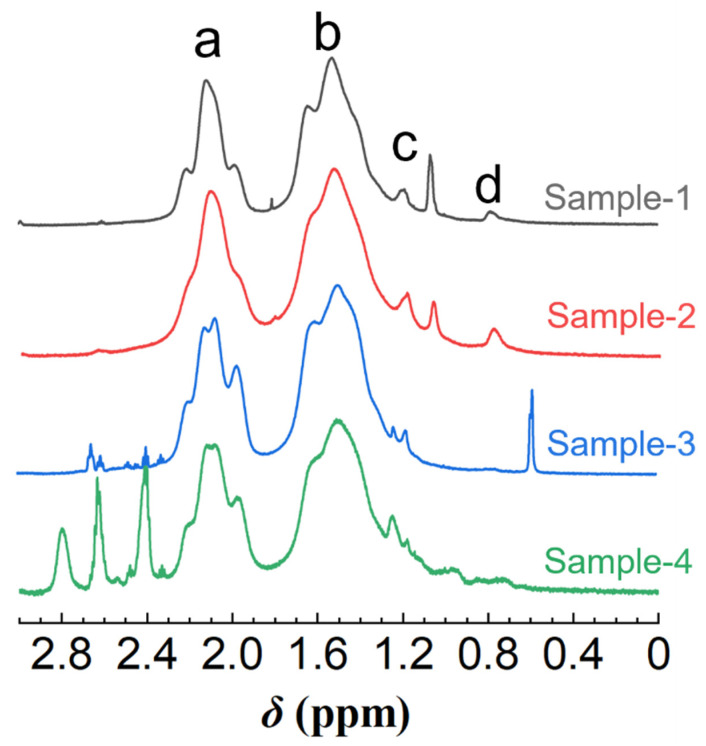
^1^H NMR spectra of four HHAPAM samples in D_2_O. (a–d refer to the proton peak in the corresponding groups).

Having validated that the ANS fluorescence probe is practicable for HHAPAM polymers, we then investigated the fitness of Equation (5). Based on the *I*/*I*_0_ values at *C*_p_ = 0.5 wt% in Figure 7, the *C*_H_ values of four polymer samples were calculated using Equation (5), i.e., 6.43 × 10^−3^, 7.95 × 10^−3^, 7.61 × 10^−3^, and 6.82 × 10^−3^ mol/L, respectively. Further determining [*H*] requires the *L* value, which was characterized by ^1^H NMR. As shown in Figure 8, four characteristic peaks (a, b, c, d) are consistent with that of HAPAM observed in Figure 1, proving that the hydrophobic groups of these HHAPAM polymers are long-chain alkyls.

The integral area of all characteristic proton peaks is listed in Table 2. According to the relationship between the ratio of protons on the corresponding groups of peaks c and d, the *L* can be calculated using Equation (6), where (*L* − 2) stands for the number of methylene groups in the alkyl chain without the terminal methyl group and the methylene group attached to the amide bond; coefficient 2 is the proton number of one methylene. Constant 3 in the denominator is the proton number of the terminal methyl group.

(6) $$\frac{2\left(L-2\right)}{3}=\frac{S_{c}}{S_{d}}$$

Consequently, the *L* values for the four polymers are 8, 8, 18, and 10 in sequence. In this way, the [*H*] values based on fluorescence spectra are separately 1.16, 1.45, 0.61, and 1.02 mol% (Table 2, column 7). On the other hand, we can compute [*H*] values of four polymers from ^1^H NMR spectra using Equations (1) and (2) (Table 2, column 6), which is surprisingly in accordance with results from the fluorescence probe method, especially for samples 1, 2, and 4.

There are two possible reasons for the uncertainty in sample 3. The first one is the inhomogeneity of the components. As previously stated, the most common synthesis method for hydrophobically associating polymers is micelle polymerization, which, despite high conversion rates, does not guarantee the uniform distribution of hydrophobic groups throughout the polymer chain. The second reason could be the spatially constrained impact of alkyl groups on the hydrophobic association. However, in terms of results, an error of about 0.09 mol% is perfectly sufficient for field testing. In other words, Equations (4) and (5) can be used to determine the hydrophobic content of hydrophobic associating polymers or polyelectrolytes after shielding the electrostatic effect.

## 3. Experimental Section

### 3.1. Materials

All chemicals and materials used as received are listed in Table 3, where four hydrophobic associating hydrolyzed polyacrylamide (HHAPAM) products were denoted as sample 1, sample 2, sample 3, and sample 4. Water (18.25 mΩ·cm) was double-deionized with an ultrapure water purification system (CDUPT-III, Chengdu Ultrapure Technology, Chengdu, China). The molecular weight of PAM is 10^7^ g/mol.

### 3.2. Synthesis of HAPAM by Post Modification

**Figure 9:**
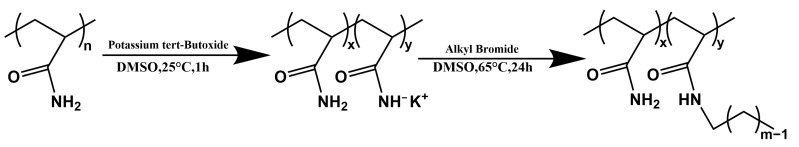
The synthetic route to prepare HAPAM.

The specific synthesis process was shown in Figure 9, and all materials and solvents are kept in the glove box before use to ensure a water-oxygen-free environment.

A typical procedure for PAM modification with octyl bromide is described as follows: 4.0 g PAM (5.63 × 10^−2^ mol AM) and 380 mL of anhydrous DMSO were introduced into a 1 L four-neck round-bottom flask equipped with a condenser, a nitrogen inlet/outlet, and a mechanical stirrer. The system was continuously stirred at 80 °C for 20 h under a nitrogen atmosphere and then cooled to room temperature when PAM in DMSO was completely dissolved. Afterwards, potassium tert-butanol (0.332 g, 2.81 × 10^−3^ mol) in 10 mL of anhydrous DMSO was added dropwise to the above PAM solution under active agitation. After stirring for 1 h, octyl bromide (0.110 g, 0.56 × 10^−3^ mol) dissolved in 10 mL DMSO was then added via a syringe dropwise. The alkylation reaction proceeded at 65 °C for 24 h while maintaining a nitrogen atmosphere and an agitation condition. The reaction was terminated by adding 400 mL cold water, and the resultant emulsion mixture was dialyzed for one week and lyophilized to obtain a white solid product for later use.

The above sample was denoted H8-x, where “H” represents the hydrophobically associating polymer, “8” stands for the *L*, and finally “x” means the [*H*]. To modulate the hydrophobicity of HAPAM, octyl bromide with different contents and 1-bromododecane and decyl bromide at the same content levels were used to prepare a series of HAPAM polymers using the same process.

### 3.3. ^1^H NMR Spectroscopy

Polymer samples were dissolved in D_2_O at a concentration of 1 wt%. The ^1^H NMR spectra were recorded on a Bruker AV II-400 MHz NMR spectrometer at 400 MHz and room temperature.

### 3.4. Fluorescence Spectroscopy

Fluorescence spectroscopy was performed on a FluoroMax-4 fluorescence spectrophotometer (HORIBA, Kyoto, Japan) at 25 °C and an excitation wavelength of 415 nm. The emission wavelength was selected as 430−630 nm. Both the excitation and emission slit widths were fixed at 2.5 nm.

Sample solutions for the ANS probe experiments were prepared as follows: A solvent containing 9.9 g/L NaCl and 0.1 g/L CaCl_2_ was first prepared using deionized water, which was used to dissolve ANS to obtain a fluorescent solvent with a concentration of 0.05 wt%. Then, a specific amount of polymer powder was slowly added to the fluorescent solution with gentle magnetic stirring for 24 h. All solutions were wrapped in tin foil and stored in a dark place before testing.

## 4. Conclusions

In summary, this paper reports a novel approach to quantitatively determine the hydrophobic content ([*H*]) of hydrophobically associating polyacrylamides (HAPAM) using a fluorescent probe—ANS. A series of HAPAMs with known structures were synthesized by chemical modification. The fluorescence spectral results indicated that the fluorescence intensity of the HAPAM/ANS solution can gradually increase upon increasing the polymer concentration, a relationship corresponding to the relative fluorescence intensity and hydrophobic content. At a fixed concentration of both polymer (0.5 wt%) and ANS (0.05 wt%), an expression between the relative fluorescence intensity of polymer solution to solvent (*I*/*I*_0_) and the molar concentration of alkyl units in hydrophobic side chains (*C*_H_) was established as *I*/*I*_0_ = 1.15 + 1.09 × 10^8^ *C*_H_^3.42^. Eventually, by combining the fluorescent probe and ^1^H NMR methods, this quantitative equation was validated to also apply to industrial hydrophobic associating hydrolyzed polyacrylamide (HHAPAM) products.

## Figures and Tables

**Table 1:** Integrated area of ^1^H NMR proton peaks and calculated hydrophobic content of all H-series polymers.

Polymer Samples	*S*_a_ + *S*_b_ + *S*_c_	*S*_d_	x (mol%)
H8-x1	387.12	1.00	0.3
H8-x2	202.38	1.00	0.5
H8-x3	149.20	1.00	0.7
H8-x4	116.52	1.00	0.9
H8-x5	102.13	1.00	1.0
H8-x6	72.30	1.00	1.5
H10-x	206.34	1.00	0.5
H12-x	199.82	1.00	0.5

**Table 2:** Comparison of hydrophobe contents ([*H*]) measured from ^1^H NMR spectroscopy to fluorescence spectra.

Sample Number	*S*_a_ + *S*_b_ + *S*_c_	*S*_c_	*S*_d_	*L* from ^1^H NMR Spectroscopy	[*H*] from ^1^H NMR Spectroscopy	[*H*] from Fluorescence Spectra
1	89.88	3.77	1.00	8	1.16	1.16
2	71.74	3.99	1.00	8	1.48	1.45
3	203.54	10.53	1.00	18	0.52	0.61
4	99.40	5.16	1.00	10	1.06	1.02

**Table 3:** Details of chemicals and materials used as received.

Chemicals or Materials	Grade	Manufacturers
8-anilino-1-naphthalenesulfonic acid (ANS)	≥95%	J&K, Shanghai, China
Potassium tert-butoxide	99%	Adamas-beta, Shanghai, China
1-bromododecane	99%	Adamas-beta, Shanghai, China
Decyl bromide	99%	Adamas-beta, Shanghai, China
Octyl bromide	99%	Adamas-beta, Shanghai, China
Anhydrous dimethyl sulfoxide (DMSO)	99.7%, water ≤ 50 ppm	Adamas-beta, Shanghai, China
Polyacrylamide (PAM)	/	Shandong Juxin New Materials, Dongying, China
Partially hydrolyzed polyacrylamide (HPAM)	/	Shandong Juxin New Materials, Dongying, China
NaCl	AR	Chengdu Chron Chemicals, Chengdu, China
CaCl_2_	AR	Chengdu Chron Chemicals, Chengdu, China
HHAPAM sample 1	/	SNF, Taizhou, China
HHAPAM sample 2	/	Beijing Hengju Polymer, Beijing, China
HHAPAM sample 3	/	Heilongjiang Jidi Oilfield Service, Suihua, China
HHAPAM sample 4	/	Sichuan Guangya Polymer Chemical, Chengdu, China

## Data Availability

The data presented in this study are available on request from the corresponding author.

## Supplementary Materials

The following supporting information can be downloaded at: https://www.mdpi.com/article/10.3390/molecules28104152/s1, Figure S1: Normalized absorption spectra at 25 °C for polymer/ANS and pure ANS solutions, with polymer concentrations of 0.2 wt% and ANS concentrations of 0.05 wt%.; Table S1: Results of degree of hydrolysis (DH) measurements for four HHAPAM polymers.

## Sample Availability

Samples of the compounds are available from the authors.
