# Supplementary material for: A Quantitative Approach to Determine Hydrophobe Content of Associating Polyacrylamide Using a Fluorescent Probe
Source: Molecules. 2023 May 17;28(10):4152. doi: 10.3390/molecules28104152 (PMC10221327; doi:10.3390/molecules28104152)
Supplement: Supplementary file 1 [file molecules-28-04152-s001.zip › molecules-2353473-supplementary.pdf]

Supporting Information *for*

A quantitative approach to determine hydrophobe content of associating polyacrylamide using a fluorescent probe

Ziyang Su[1], Yu Zhang[2], Weidong Liu[2], Ruijing Han[2], Xuezhi Zhao[1], Xiaohuo Shi[3], Xingyu Lu[3], Yan Zhang[1]*, Yujun Feng[1]*

[1] Polymer Research Institute, State Key Laboratory of Polymer Materials Engineering, Sichuan University, Chengdu 610065, People's Republic of China; szy@stu.scu.edu.cn (Z. S.); zhaoxz@scu.edu.cn (X. Z.); y.zhang@scu.edu.cn (Y. Z.); yjfeng@scu.edu.cn (Y. F.)

[2] Research Institute of Petroleum Exploration & Development , PetroChina Company Limited , Beijing 100083, People's Republic of China; zhangyu01@petrochina.com.cn (Y. Z); lwd69@petrochina.com.cn (W. L.); hanrj_18@petrochina.com.cn (R. H.)

[3] Instrumentation and Service Center for Molecular Sciences, Westlake University, Hangzhou 310024, People's Republic of China; shixiaohuo@westlake.edu.cn (X. S.); luxingyu@westlake.edu.cn (X. L.)

* Correspondence: y.zhang@scu.edu.cn (Y. Z.); yjfeng@scu.edu.cn (Y. F.)

S1. Measurement of the degree of hydrolysis ($\overline{DH}$) for four HHAPAM Samples

The degree of hydrolysis (DH) of polymers was tested by acid-base titration, and the test procedure referred to the work of Chen et al.[1]. The DH value was calculated according to the following equation:

$$DH=\frac{71\times C\times V}{m-23\times C\times V}\times 100 \tag{S1}$$

where C stands for the concentration of HCl standard solution, mol/L; V represents the volume of the HCl standard solution consumed by the sample solution, mL; m signifies a mass of 0.1 wt% sample solution, g; 23 means the difference in mass of sodium acrylate and acrylamide unit, g/mol; and 71 stands for the mass of the acrylamide chain equivalent to the 1.00 ml HCl standard solution [C (HCl) = 1.000 mol/L], g/mol.

The results of four polymer samples are shown in **Table S1**, which confirms that all samples are hydrophobic associating polyelectrolytes:

Table S1. Results of degree of hydrolysis ($\overline{DH}$) measurements for four HHAPAM polymers

Sample number	Group	*m* (g)	*V* (mL)	DH (%)	$\overline{DH}$ (%)
	1	29.9881	8.8	22.3	
1	2	30.0024	8.9	22.6	22.6
	3	30.0363	9	22.9	
	1	29.9851	9.3	23.7	
2	2	30.0572	9.3	23.7	23.5
	3	29.9711	9.1	23.2	
	1	30.0606	7.8	19.6	
3	2	30.1522	7.8	19.5	19.6
	3	30.0485	7.8	19.6	
	1	29.9855	9.3	23.7	
4	2	30.0576	9.3	23.7	23.5
	3	29.9715	9.1	23.2	

S2. Measurement of absorption spectra by UV absorption spectrometry

To analyze the variation in ANS absorption in polymer solutions, we took one HAPAM sample (H8-1.0) and one HHAPAM polymer (sample-1) as examples. All sample solutions were tested by a UV-6100 spectrophotometer (MAPADA, Shanghai) at 25 °C.

Figure S1 depicts the normalized absorption spectra for polymer/ANS and pure ANS solutions, with polymer concentrations of 0.2 wt% and ANS concentrations of 0.05 wt%. It was found that the maximum absorption wavelength of ANS is almost unchanged, demonstrating that the ground state of ANS was unaffected with the addition of the polymer. In this case, the changes in the fluorescent emission spectra of the polymer/ANS solutions at a fixed excitation wavelength can be used to reflect the polymer-induced microenvironment variation.

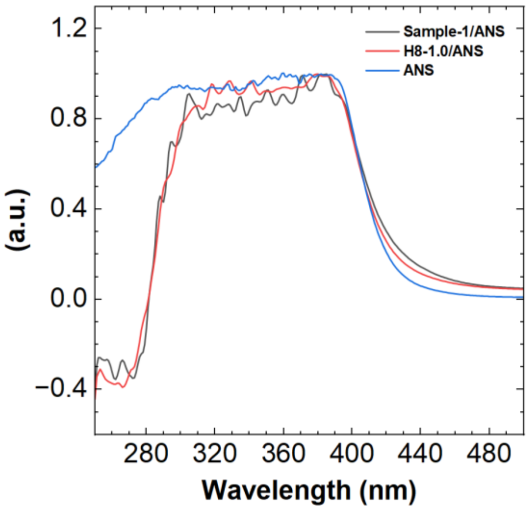

Figure S1. Normalized absorption spectra at 25 °C for polymer/ANS and pure ANS solutions, with polymer concentrations of 0.2 wt% and ANS concentrations of 0.05 wt%.

References

1. Chen, C.; Sun, L.; Liu, X. Effect of degree of hydrolysis of polyacrylamide on the micromorphology of its solution. *J. Appl. Polym. Sci.* **2022**, *139*.
